# Tumor Microenvironment and Immunotherapy-Based Approaches in Mantle Cell Lymphoma

**DOI:** 10.3390/cancers14133229

**Published:** 2022-06-30

**Authors:** Khalil Saleh, Morgane Cheminant, David Chiron, Barbara Burroni, Vincent Ribrag, Clémentine Sarkozy

**Affiliations:** 1 Department of Hematology, Gustave Roussy Cancer Campus, 94805 Villejuif, France; khalil.saleh@gustaveroussy.fr (K.S.); vincent.ribrag@gustaveroussy.fr (V.R.); 2 Department of Hematology, Necker Hospital, Assistance Publique des Hopitaux de Paris (APHP), 75004 Paris, France; morgane.cheminant@aphp.fr; 3 Nantes Universite, Inserm, CNRS, Universite d’Angers, CRCI2NA, 44007 Nantes, France; david.chiron@univ-nantes.fr; 4 Department of Pathology, Cochin Hospital, Assistance Publique des Hopitaux de Paris (APHP), 75004 Paris, France; barbara.burroni@aphp.fr; 5 Departement d’Innovation Therapeutique et d’Essais Precoces (DITEP), Gustave Roussy Cancer Campus, 94805 Villejuif, France

**Keywords:** mantle cell lymphoma, tumor microenvironment, SOX11, Bcl2, Bruton’s tyrosine kinase, BAFF, BCR, NF-kB

## Abstract

**Simple Summary:**

Mantle cell lymphoma (MCL) is associated with poor response to anthracycline-based chemotherapy, and high-dose cytarabine offers a durable response. Recently, multiple targeted therapies have been approved for the treatment of MCL, highlighting the role of the tumor microenvironment (TME) in the expansion and resistance of the disease. We review herein the TME in MCL and the different therapeutic strategies of treatment.

**Abstract:**

Mantle cell lymphoma (MCL) is an aggressive B-cell non-Hodgkin lymphoma (NHL) characterized by the translocation t(11;14) (q13;q32) and a poor response to rituximab–anthracycline-based chemotherapy. High-dose cytarabine-based regimens offer a durable response, but an important number of MCL patients are not eligible for intensive treatment and are ideal candidates for novel targeted therapies (such as BTK, proteasome or BCL2 inhibitors, Immunomodulatory Drugs (IMiDs), bispecific antibodies, or CAR-T cell therapy). On the bench side, several studies aiming to integrate the tumor within its ecosystem highlighted a critical role of the tumor microenvironment (TME) in the expansion and resistance of MCL. This led to important insights into the role of the TME in the management of MCL, including potential targets and biomarkers. Indeed, targeted agents often have a combined mechanism of action on the tumor B cell but also on the tumor microenvironment. The aim of this review is to briefly describe the current knowledge on the biology of the TME in MCL and expose the results of the different therapeutic strategies integrating the TME in this disease.

## 1. Introduction

Mantle cell lymphoma (MCL) represents 5 to 7% of malignant lymphomas with an annual incidence rate of 1 to 2 per 100,000 [1,2,3]. Historically, MCL was associated with a dismal prognosis, with a median overall survival (OS) ranging from 3 to 5 years [4]. In the past 10 years, tremendous progress has been made in the standard of care for these patients, using high-dose cytarabine followed by rituximab maintenance as first-line or using novel targeted agents in the relapse setting, leading to drastic improvement in the progression-free survival (PFS) and OS. On the bench side, the biology of MCL is now better characterized, leading to recent updates in the WHO classification. Indeed, MCL is now well recognized as a heterogeneous disease with two main clinical and biological presentations belonging to two distinct categories: nodal MCL, which represents the majority of patients (classical MCL, cMCL 80–90%) and leukemic non-nodal MCL (nnMCL, 10–20%), which can be distinguished based on sex-determining region Y-box 11 (SOX11) expression, immunoglobulin heavy chain variable region (IGHV), mutation load, and clinical behavior [5]. More recently, a recent genomic and transcriptomic integrated analysis of more than 150 samples classified MCL in four main molecular subgroups associated with outcome [6].

These heterogeneous clinical presentations and biological features led to various treatment modalities, mostly relying on age and disease subtype in the first-line setting [7].

Several targeted therapies have been approved by the Food and Drug Administration (FDA) and the European Medicine Agency (EMA) for the treatment of relapsed/refractory (R/R) MCL. Recent bench side discoveries highlighted their role on the tumor microenvironment (TME) in MCL, making TME a key player in MCL. Within these targeted therapies, some of them target the B-cell receptor (BCR), such as Bruton’s tyrosine kinase inhibitor (BTKi) (ibrutinib [8,9], acalabrutinib [10], and zanubrutinib [11]), the proteasome such as bortezomib [12], mammalian target of rapamycin (mTor) with temsirolimus [13,14], or Cereblon (CRBN) through the use of lenalidomide [15]. In addition to the well-known action of lenalidomide as an immunomodulatory agent (IMIDs) aiming at fostering the immune system [15], the role of BTK on the TME has also been depicted recently. Indeed, ibrutinib induced the redistribution of chronic lymphocytic leukemia (CLL) or MCL cells from the lymph node microenvironment to the peripheral blood. It also exerts immunomodulatory effects through regulation of tumor-infiltrating macrophages [16,17,18]. More recently, brexucabtagene autoleucel (KTE-X19), an anti-CD19 chimeric antigen receptor (CAR)-T cell therapy [19], but also bispecific antibodies (BsAb) [20], showed impressive results in third line.

In the present review, we aim to discuss the main biological characteristics of MCL, and more particularly of the TME leading to biological rational for immune checkpoint (ICP) inhibitors, BsAb, IMIDs, and CAR-T cell therapy, and present the safety and efficacy of the main clinical trials in this setting.

## 2. The Biology of Mantle Cell Lymphoma’s Tumor Microenvironment

MCL is characterized by the presence of the chromosomal translocation t(11;14) (q13;q32), considered as the genetic hallmark and one of the primary oncogenic events [21,22]. However, this event is not sufficient to confer the full lymphoma phenotype. MCL is also characterized by a high genomic instability with a high number of secondary genetic alterations involving cell cycle regulation, DNA damage response, cell death, NF-kB, or epigenetic modifiers, with a median number of six secondary events [23,24,25]. ATM, CCND1, TP53, and RB1 are among the most recurrently mutated genes [26]. These alterations are not further described here as this is not the topic of the review.

The molecular landscape of cMCL versus nnMCL subtypes has recently been depicted in a combined analysis of whole genome sequencing, transcriptome, and DNA methylome, by Nadeu and colleagues. The authors found that the mutational burden was identical between the two subtypes but cMCL had a significantly higher number of structural and complex variants than nnMCL, in particular breakage fusion bridge cycle, copy number alterations, and driver alterations. ATM alterations were exclusively found in cMCL versus CCND1 SHM largely enriched in nnMCL in relation with a post-GC B cell of origin [23,27].

In addition to cell-intrinsic anomalies, it is now well accepted that the tumor must be studied as an ecosystem by integrating the multiple dialogs occurring between the tumor and its TME. Recently, several fundamental and translational studies were conducted to decipher the composition of the TME and to understand its crosstalk with MCL cells and its potential therapeutic function. A dynamic interplay between tumor B and TME cells within the lymph node (LN), leading to cell cycle activation, apoptosis inhibition, and drug resistance through NF-kB and BCR activation, has been reported [28,29,30]. Furthermore, proliferation of MCL cells is more important in LN than in circulating tumor cells, independently of tumor intrinsic abnormalities, and in correlation with B-cell receptor (BCR) and NF-kB activations [29].

### 2.1. BCR Activation in the Lymph Nodes versus Peripheral Blood MCL Cells

MCL cell survival relies on BCR-mediated signaling and NF-kB pathways [31,32]. Several studies assessed the role of the TME interactions with the tumor B cell in this context, and subsequently the impact of BTK inhibitors [21,29,32]. Indeed, it is now well known that selective inhibition of BTK (or PI3Kdelta) within the BCR (or CXCR4) pathways leads to peripheral lymphocytosis by disrupting the interaction between the TME and tumor B cell and their homing [8,17,33,34].

Using MCL samples collected from LN and peripheral blood, Saba and colleagues demonstrated that, in vivo, gene expression profiles differed between MCL cells in peripheral blood and in LN. This was mostly due to activation of the BCR and NF-kB signaling pathways, specifically in LN-resident MCL cells. The TME, per se absent in peripheral blood tumor cells, has therefore a clear role in proliferation of LN-MCL cells [29].

### 2.2. CD40-CD40 Ligand Axis

As observed in several mature B-cell malignancies (i.e., CLL, follicular lymphoma (FL)), the interaction between CD40 on the surface of tumor cells and its ligand CD40L contribute to MCL cell proliferation and viability [35].

Further deciphering the central role of the TME using ex vivo culture models, Chiron et al. showed that lymphoid-like signals (CD40L+ cells) induced proliferation of primary MCL cells. The cell cycle progression was further amplified by specific MCL cytokines (IGF-1, BAFF, IL-6, IL-10), selected based on cytokine receptor expression by the tumor in situ. In contrast, whereas stromal cells protected MCL cells against spontaneous apoptosis, they were not able to promote robust proliferation in this model. Based on integrated transcriptomic and functional analysis, they showed that peripheral blood MCL cells cultured in the presence of CD40-L and cytokine stimuli displayed cellular (proliferation, survival) and molecular (NF-kB pathways, Bcl-2 family, secretome) profiles similar to the ones seen in lymph-node-resident MCL cells, emphasizing the relevance of the model and the role of CD40-L expressing T cells for tumor survival [28,33,36]. Further characterization of MCL TME in situ is now needed to characterize these T-cell populations.

Based on the same ex vivo culture model (CD40-L and cytokines) designed to mimic signals occurring in the LN, Chiron et al. also showed that proliferating MCL cells had an imbalance in the expression of the Bcl-2 family, leading to the loss of mitochondrial priming. This loss of mitochondrial priming happened through the induction of anti-apoptotic proteins, especially Bcl-XL, associated with a decrease in proapoptotic proteins (Bim, Bax, and Bak). This led to a resistance to Bendamustine (alkylating agent) and venetoclax (BH3 mimetics targeting Bcl-2), but not Bortezomib (proteasome/NF-kB inhibitor). This CD40-L/NF-kB-dependent upregulation of Bcl-XL could be counteracted by obinutuzumab, a type II anti-CD20 monoclonal antibody [28] expressing a high level of CD20 in MCL. Accordingly, obinutuzumab (but not rituximab) overcame the loss of priming through the inhibition of the NF-kB/Bcl-XL axis, and consequently sensitized cells to venetoclax in vitro. Importantly, in the AIM trial combining Ibrutinib and venetoclax, in vivo resistance was also associated with an overexpression of Bcl-XL induced by abnormalities in the SWI-SNF pathway (SMARCA2 or SMARCA4 mutations), leading to downregulation of ATF3, a direct Bcl-XL repressor [37]. These preclinical findings, in line with other reports [33,38], provided a rationale of a combination targeting both tumor B cell and TME with obinutuzumab, ibrutinib and venetoclax, detailed later [39].

### 2.3. Monocytes/Macrophages and Dynamic Interaction between MCL Cells and Myeloid TME

In addition to the lymphoid TME, the myeloid ecosystem plays a central role in numerous cancer types. In MCL, recent studies have shown that LN-infiltrating MCL-associated macrophages correlate to a poor prognosis [40], suggesting that targeting the MCL/macrophage dialog could be a perspective of interest to develop innovative therapeutic options.

Ex vivo, Papin and colleagues showed that the co-culture of primary MCL cells with monocytes contributed to their differentiation into adherent macrophages, supporting MCL cell proliferation and survival up to several months after culture. They also demonstrated that adherent macrophages have an M2-like phenotype characterized by the expression of CD163 and a specific protumoral secretome. This polarization was mediated by an MCL-specific secretion of CSF1, and to a lesser extent IL-10, which could be abrogated with ibrutinib, highlighting here again the predominant role of the TME in the ibrutinib mechanism of action in MCL [41]. Nevertheless, this inhibition was ineffective in ibrutinib-resistant samples, leading to the rationale of using CSF1R inhibitors to block the MCL/macrophage dialog, as currently tested in several solid tumors [41].

More recently, Decombis et al. demonstrated that MCL cells also present a tumor-specific secretion of interleukin-32 beta (IL32β) through CD40-L-mediated interaction with the TME, but also IL32 locus hypomethylation. This secretion is dependent on the alternative NF-κB pathway (NF-kB2) and is able to differentiate monocytes into specific CD163 macrophages, supporting MCL cell survival through a soluble dialog mostly driven by BAFF (a member of the tumor necrosis factor (TNF) family), making the IL32/BAFF axis a novel key target to be disrupted, potentially targeting the NIK/NF-kB2 pathway [36]. The role of the BAFF/BAFF-R axis in the pathogenesis of MCL has also been supported in other studies [42,43,44,45].

### 2.4. Stromal Cells

In several B-cell malignancies, stromal cells have a key role in triggering the activation of numerous signaling pathways (BCR, PI3K/AKT, JAK/STAT, and NF-kB) through chemokine receptors and adhesion molecules critical for malignant B-cell trafficking and homing, leading to drug resistance [46,47]. In MCL, several reports showed that mesenchymal stromal cells contribute to the survival and proliferation of primary MCL cells, as well as protection from drug-induced apoptosis.

The adhesion of MCL tumor cells to stromal cells is attributed in part to the high level of expression of functional CXCR4, CXCR5 chemokine receptors, and VLA-4 adhesion molecules on the surface of MCL [47,48,49]. In vitro, migration of MCL cells beneath bone marrow stromal cells conferred drug resistance, which could be blocked by a CXCR4 antagonist (plerixafor) and VLA-4 antibody (natalizumab) [47]. The contribution of CXCR4 silencing to the reduction in proliferation, cell adhesion to bone marrow stroma cells, and colony formation of MCL cells has also been reported by Chen et al. In vivo, the presence of quiescent MCL cells in the bone marrow was also reduced by CXCR4 silencing, whereas the co-culture of MCL cells with human bone marrow stromal cells, or SDF-1, led to markedly increased MCL colony formation [50].

Focal adhesion kinase (FAK) is a novel “stromal”-related therapeutic target as a major signaling molecule, highly expressed in MCL, that functions downstream of integrins (including CXCL12) and that translates signals from the extracellular matrix in the bone marrow. To study more in detail the role of FAK in MCL, Rudelius et al. used a co-cultured model of MCL cell lines and bone marrow stromal cells (BMSC) with small inhibitors of FAK [32]. The co-culture of BMSC led to the activation of FAK signaling in primary MCL cells and MCL lines with activation of pro-survival pathways (AKT, NF-kB). The inhibition of FAK led to the blockade of cell invasion and induced apoptosis via inactivation by suppression of several pathways such as classical and alternative NF-kB. A synergy was found between FAK and BTK inhibition, and ibrutinib resistance could be overcome with FAKi [32].

Medina and colleagues demonstrated in an ex vivo co-culture system in which the interaction between MCL and stromal cells led to the activation of the BAFF/NF-kB axis, which induced drug resistance and increased CXCL12- and CXCL13-mediated cell migration [51]. The identification of BAFF in the dialog with stromal cells, but also with monocytes/macrophages as previously mentioned, make this factor a central player in the MCL ecosystem. More recently, Zhang and colleagues reported that the knockdown of BAFF-R contributed to MCL cell death. Conversely, the addition of recombinant BAFF protected MCL cells from cytarabine-induced apoptosis. Finally, the authors demonstrated both in vitro and in vivo the efficacy of a humanized defucosylated antibody-dependent cell cytotoxicity (ADCC) optimized anti-BAFF-R antibody in killing MCL [52].

### 2.5. SOX11 and MCL TME Composition and Modulation

SOX11 is a neural transcription factor identified as a highly specific marker for both cyclin-D1-positive and -negative MCL [53], not detected in other B-cell malignancies or normal lymphoid cells, and discriminatory between the two previously described clinical entities of MCL [54]. SOX11 regulates key transcriptional programs such as mature B-cell differentiation, modulation of the cell cycle, apoptosis, or stem cell development. In MCL, the role of SOX11 in tumor growth and development has initially been mainly related to the terminal B-cell differentiation blockade, PAX5 being one of key targets of SOX11, whose silencing induces BLIMP1 expression and plasmacytic differentiation [55].

Later, the role of SOX11 in the TME composition and modulation in MCL was highlighted. Indeed, it has been reported that SOX11-positive xenograft and human primary MCL tumors overexpressed angiogenic signatures with a higher microvascular density. Platelet-Derived Growth Factor A (PDGFA), overexpressed in SOX11+ MCL, was identified as a SOX11 direct target, whose inhibition altered the pro-angiogenic effect of SOX11 on endothelial cells [56,57].

Balsas and colleagues directly linked SOX11 expression to cell migration and stromal interaction in MCL cells, making this pathway a probing strategy to overcome stromal-mediated treatment resistance in MCL. Indeed, they showed that SOX11 directly upregulated the expression of CXCR4 and PTK2, encoding for FAK, leading to the activation of the ERK1/2 FAK and PI3K/AKT downstream pathways. This led to an increased cell migration, adhesion to stromal cells, and cell proliferation with a potential greater resistance to conventional treatment in SOX11+ MCL. Moreover, specific FAK and PI3K inhibitors reduced SOX11-mediated cell migration and stromal interactions with a reversion of cell-adhesion-mediated drug resistance to the same level of SOX11-negative MCL. In xenograft models, FAK and CXCR4 inhibitors impaired SOX11+ MCL cell engraftment in the bone marrow [58]. Based on a transcriptomic analysis combined with IHC, the authors then looked in more detail into the correlation between SOX11 expression and TME composition in MCL. They showed that SOX11+ MCL samples had a significantly lower immune infiltration and downmodulation of gene sets involved in an effective anti-tumoral immunity (assessed by the Nanostring PanCancer Immune Profiling Panel) [59]. More particularly, CD4 T cells, as well as granzyme B+ cytotoxic T cells by IHC, were less frequent in SOX11+ tumors. On the other side, several B-cell genes were upregulated in SOX11+ tumors, such as CD79A, CD19, PAX5, or BLNK and CD70. The CD70 locus was indeed identified as a direct target of SOX11, and could be induced thanks to CD40-L stimulation, part of the tumor B/TME interaction. The expression of CD70 was correlated with blastoid morphology, Ki67 scoring, and a poor OS, independently from TP53 alterations and tumor cell morphology. Finally, CD70 expression was associated with T-reg cell infiltration, characterized by FOXP3 and CTLA4 IHC expression, which was greater in SOX11+ MCL samples [59].

### 2.6. Immune Checkpoint Expression in MCL

As opposed to other specific lymphoma subtypes, the expression and the role of ICP molecules, such as programmed death ligand-1 (PD-L1) in MCL remains controversial, as the early trials of immunotherapy were not conclusive. In a co-culture model of allogenic T cells and MCL cells, Wang et al. demonstrated that PD-L1 expression on MCL cells was able to inhibit T-cell proliferation induced by tumor cells and impaired the generation of antigen-specific T-cell responses. Blocking PD-L1 on MCL cells enhanced T-cell responses and tumor cell killing in vivo. These MCL-reactive T cells were memory effector T cells with high expression of perforin, granzyme B, and CD107a and interferon γ secretion [60]. The possible role of PD-L1 in vitro was reported in another co-culture study that showed an induction of PD-L1 expression through CD40/CD40-L interaction, whereas PD-L2 expression was not observed. Interestingly, this expression could be attenuated by BTK or PI3K inhibition [61]. However, in this report, PD-1 expression on CD8+ cells from MCL patients was comparable to the one from healthy donors. Importantly, the absence of PD-L2, or PD-1 expression in MCL biopsies, was confirmed by others that furthermore did not find a significant PD-L1 overexpression [62].

## 3. TME as a Therapeutic Option in MCL?

We present here the rationale and results of key recent clinical trials using drugs modulating the TME in MCL, such as pathway inhibitors, ICP, IMIDs, CAR-T, or BsAb.

### 3.1. The BTKi and BCL2i Combination to Overcome TME-Related Resistance

BTKi are approved for the treatment of R/R MCL. Ibrutinib was associated with an objective response rate (ORR) of 68% [8], acalabrutinib 81% [10], and zanubrutinib of 84% [11]. BTKi modulate the TME and tumor B-cell interaction, leading to a redistribution of lymphocytosis caused by inhibition of signaling and function of chemokine receptors (CXCR4, CXCR5) and adhesion molecules. In a phase I trial including 28 patients with R/R MCL, the Bcl-2 inhibitor venetoclax was associated with an ORR of 71% [63]. Bcl-2 inhibition resistance has been related to Bcl-XL overexpression that can be overcome thanks to a BTK inhibition combination. Indeed, mobilized MCL cells after BTK inhibition express less Bcl-XL than MCL cells within reactive lymph nodes (RLN). This can be explained by the role of the CD40/CD40-L interaction within the lymph node TME in Bcl-XL expression induction. Therefore, these mobilized MCL cells are more sensitive to Bcl-2 inhibition [33].

Therefore, the combination of venetoclax (increasing doses to 400 mg per day) and ibrutinib (560 mg per day) has been evaluated in the phase II AIM trial in patients with MCL. Overall, 24 patients were treated, including one patient with previously untreated disease. The CR rate at week 16 according to computed tomography was 42% and 62% using PET-CT. The minimal residual disease (MRD) was evaluated using flow cytometry, and 67% of patients achieved MRD negativity. The estimated 12-month PFS and OS rates were 75% and 79%, respectively [64].

Based on the rationale previously exposed, the combination of BTKi, BCL2i, and obinutuzumab was evaluated in the OASIS phase I/II trial in patients with untreated or relapsed MCL [39]. A total of 48 patients were enrolled, including 15 untreated patients. A dosage of 400 mg of venetoclax was chosen for the expansion cohort. The CR rate evaluated using PET-CT at the completion of cycle 6 was 86.6% (13/15) in untreated patients and 70% (23/33) in relapsed patients. For relapsed patients, the 2-year PFS rate was 69.5% and the OS rate was 68.6%, and for untreated patients, the 1-year PFS rate was 93.3%. Moreover, for MRD-evaluable patients, 100% (12/12) of untreated patients achieved MRD negativity, and MRD clearance was seen in 71.5% (10/14) of relapsed patients. The combination was associated with acceptable safety, with grade 3 to 4 adverse events occurring in 75% of relapsed patients and 53% of previously untreated patients [39], leading to the phase II trial that is currently recruiting (NCT04802590).

### 3.2. Immunomodulatory Agents, Lenalidomide

#### 3.2.1. Rationale of IMIDs in MCL and Potential Mechanism of Action

Lenalidomide is a second generation of Thalidomide Analogues with a broad antineoplastic and antiproliferative mechanism of action, as well as TME modulation through CRBN, the molecular target of these drugs. Lenalidomide is approved for the treatment of B-cell malignancies such as multiple myeloma (MM), FL, and MCL [15,65].

Indeed, it is now well shown that IMIDs target CRBN, leading to the degradation of key neo-substrates such as IKAROS and AIOLOS, but also ZMYM2 (ZNF198), a transcription factor involved in balanced chromosomal rearrangements with FGFR1 and FLT3 [66]. Given its action via CRBN, lenalidomide has a direct cytotoxic effect against neoplastic cells, but also a strong impact on the peripheral immune system as well as the TME by immunomodulating and fostering the activity of T and NK and downregulating T-reg and myeloid-associated tumor cells, such as M2 macrophages [67,68,69,70,71,72,73]. Furthermore, in preclinical models of non-Hodgkin lymphoma (NHL), lenalidomide enhanced the ADCC of rituximab, which was the rationale of the combination of the two drugs in NHL [71,74].

#### 3.2.2. Clinical Data of Lenalidomide in R/R MCL

Lenalidomide (25 mg orally per day D1–21 every 28 days) was initially evaluated as a single agent in the MCL-001 (EMERGE) phase II trial in patients with MCL who relapsed or progressed after or were refractory to bortezomib. The ORR was 28% (37/134 patients) with a median PFS of 4.0 months, and median OS of 19.0 months [75]. The MCL-002 (SPRINT) phase II trial randomized patients with R/R MCL to receive either lenalidomide or the investigator’s choice treatment. Lenalidomide was associated with significantly longer PFS compared with investigator’s choice at a median follow-up of 15.9 months (8.7 months versus 5.2 months, HR, 0.61; 95% CI, 0.44–0.84; *p* = 0.004) [15]. These data led to the FDA approval of lenalidomide for the treatment of R/R MCL.

Lenalidomide has also been evaluated in combination in multiple phase I/II trials (Table 1). In the PHILEMON phase II trial, lenalidomide in combination with rituximab and ibrutinib was tested, and the ORR was 76% (38/50) including 56% of CR with a median OS of 22 months [76]. The combination of Venetoclax, ibrutinib, prednisone, obinutuzumab, and lenalidomide (ViPOR) is currently under investigation in a phase Ib/II trial in patients with R/R and untreated MCL (NCT03223610), and the ORR was 100% with 80% of CR.

#### 3.2.3. Lenalidomide in the First-Line Setting

In the upfront setting, the combination of lenalidomide and rituximab showed an ORR of 92% with 64% of CR and 5 years PFS of 64% [77]. Given the efficacy of bendamustine in MCL, the combination of lenalidomide, bendamustine, and rituximab was evaluated for elderly or unfit patients in a phase I/II trial. However, this trial highlighted toxicity issues [78].

The combination of enalidomide, rituximab, and venetoclax in patients with previously untreated MCL was evaluated in a phase Ib trial (NCT03523975). The majority of patients had stage IV disease (96%). This combination was associated with a high ORR of 96% (27/28 patients) and CR/CRu (unconfirmed CR) of 89%. Interestingly, 71% of patients had negative MRD at 10-6 sensitivity [79].

Finally, an MCL R2 elderly phase III trial evaluated the role of lenalidomide as maintenance therapy in unfit patients. In this trial, patients had a double randomization, first for induction between alternating RCHOP/RHAD for a total of six cycles versus standard RCHOP for eight cycles and for maintenance between lenalidomide in combination with rituximab (R2) versus rituximab alone for 24 months. Ribrag and colleagues recently reported the positive results of the primary endpoint for the maintenance randomization (PFS). R2 maintenance was associated with significantly prolonged PFS in comparison with rituximab alone (*p* = 0.0003) with a 2-year PFS of 76.6% in the R2 arm versus 60.8% in the rituximab arm. The OS was not statistically different between the two arms. The R2 maintenance was associated with more toxicity such as neutropenia, respiratory tract infection, and skin cancer [80]. A secondary objective of the MCL R2 elderly trial was the impact of the maintenance therapy on the prognostic value of the MRD after the induction treatment. MRD at the end of induction was available for 401 patients and MRD+ was not statistically different between the two induction regimens (42.2% (81/192) in the R-CHOP arm vs. 36.3% (76/209) in the R-CHOP/R-HAD arm, *p* = 0.23). Interestingly, in the rituximab arm, the 2-year PFS was not statistically different between MRD− and MRD+ patients (64.8% vs. 61.7%, respectively). However, in the R2 arm, MRD did have a prognostic value: the 2-year PFS was 84.3% for MRD− patients vs. 61.6% for MRD+ patients, respectively (HR: 3 (1.78–5.1), *p* < 0.0001) [81]. These results provide a potential biomarker for the use of R2 as a maintenance strategy for elderly MCL patients who present a negative MRD after induction.

**Table 1:** Lenalidomide combinations in MCL.

Agents	Indication	Phase	N of Patients	ORR (%) CR (%)	mPFS (Months)	mOS (Months)	Grade ≥ 3 Toxicities (%)
**Lenalidomide monotherapy (EMERGE study)** [75]	R/R MCL after bortezomib	II	134	28 7.5	4.0	19.0	Neutropenia (43) Thrombocytopenia (28) Anemia (11) Pneumonia (8)
**Lenalidomide monotherapy (NHL-003)** [82]	R/R MCL	II	57	35 12	8.8	NR	Neutropenia (46) Thrombocytopenia (30) Anemia (13)
**Lenalidomide vs. IC (MCL-002; SPRINT)** [15]	R/R MCL	II randomized	254 (170 vs. 84)	40 vs. 11 5 vs. 0	8.7 vs. 5.2	27.9 vs. 21.2	Neutropenia (44) Thrombocytopenia (18) Anemia (8)
**Lenalidomide + rituximab** [83]	R/R MCL	II	44	57 36	11.1	24.3	Neutropenia (36) Lymphopenia (36) Leucopenia (30) Thrombocytopenia (23) Anemia (2)
**Lenalidomide + ibrutinib + rituximab (PHILEMON)** [76]	R/R MCL	II	50	76 56	16	22	Neutropenia (38) Infections (26) Cutaneous (14) Gastrointestinal (12) Thrombocytopenia (12)
**Lenalidomide + rituximab + bendamustine (MCL4; LENA-BERIT)** [78]	Front-line MCL in unfit patients	I/II	51	88 64	42	3-year OS 73%	Neutropenia (75) Infections (42) Thrombocytopenia(20) Rash (18)
**Lenalidomide + rituximab** [77,84]	Front-line	II	38	92 64	NR 7-year PFS rate: 60.3%	NR 7-year OS rate: 73.2%	Neutropenia (50) Rash (29) Thrombocytopenia (13) Anemia (11) Tumor flare (11)
**Lenalidomide + rituximab + venetoclax** [79]	Front-line	Ib	28	96 89	NA	NA	Neutropenia (68) Thrombocytopenia (50)
**Lenalidomide + rituximab** [80]	Maintenance after first-line treatment	III	495	NA	2-year PFS: 76.6% vs. 60.8%	2-year OS: 87.3% vs. 85.8%	Neutropenia (50 vs. 18.8%) Respiratory tract infection (5.5 vs. 0.8%) Skin cancer (5.5 vs. 2.0%)
**Lenalidomide + venetoclax + ibrutinib + prednisone + obinutuzumab** [85]	R/R or untreated MCL	Ib/II	11	100 80	NR	NR	Hypokalemia (33) Neutropenia (13) Anemia (11) Thrombocytopenia (9)
**Lenalidomide + obinutuzumab (NCT01582776)**	R/R MCL	II	13	46.2 15.4	NA	NA	Infections (12.5)

IC: investigator’s choice; NR: not reached; NA: not available; ORR: objective response rate; mPFS: median progression-free survival; mOS: median overall survival; R/R: relapsed/refractory; MCL: mantle cell lymphoma; N: number.

#### 3.2.4. Translational Studies of Lenalidomide in MCL: A Biomarker of Response?

In MCL, Hagner and colleagues reported that lenalidomide increased NK-cell-mediated cytotoxicity against neoplastic cells in preclinical models through secretion of granzyme B and formation of lytic NK cell immunological synapses. Consequently, patients who responded to lenalidomide had a significantly greater increase in CD56+ NK cells relative to total lymphocytes compared to non-responders [86].

Thus far, no specific predictive markers of response to lenalidomide could be found for MCL patients. Baseline expression of its molecular target, CRBN, as well as genetic mutations reported to impact clinical response to the BTK inhibitor ibrutinib were not associated with outcome in the MCL-002 trial [86]. In other NHL subtypes or in multiple myeloma, peripheral blood T-cell composition or a specific gene expression signature has been linked with outcome after treatment with Thalidomide Analogues. However, so far, these findings have not been confirmed and are not routinely applicable [73,87]. In the MCLR2 trial, the benefit of Revlimide–rituximab maintenance over rituximab alone was observed only in patients with a negative MRD at the end of induction, offering a potential predictive marker that could be routinely available [81]. Finally, in MM CRBN expression, methylation and mutations have been associated with secondary resistance [88,89].

### 3.3. Checkpoint Inhibitors

Preclinical studies on PD-1 or PD-L1/2 expression in MCL are controversial, as exposed earlier. Therefore, the limited activity of anti PD1/PD-L1 as a single agent in these patients is not so surprising. Lesokhin and colleagues reported the results of a phase I trial evaluating nivolumab, a PD-1 inhibitor, in patients with refractory B-cell lymphoma subtypes including MCL. A total of 81 patients were enrolled in this trial, among whom only 4 patients had MCL. No significant clinical activity was observed with three patients of four who experienced stable disease as the best response to nivolumab. The toxicity profile was fully comparable in MCL patients in comparison to patients with other histology. Noteworthy, three patients of four had negative expression of PD-L1 and PD-L2, and the remaining patient had low PD-L1 positivity of around 5% [90].

Given the absence of efficacy as single agents, ongoing trials are evaluating potential combination strategies in MCL (Table 2). For example, the safety and the preliminary efficacy of pembrolizumab, in combination with ibrutinib, is under investigation in a phase I/II trial (NCT03153202), as well as the combination of nivolumab and lenalidomide (NCT03015896). Durvalumab, a programmed death ligand-1 (PD-L1) inhibitor, was evaluated in combination with loncastuximab tesirine (ADCT-402) in patients with R/R DLBCL, follicular lymphoma, and MCL. The trial was terminated with a limited number of patients because no additional evident activity was shown with the combination versus ADCT-402 monotherapy (NCT03685344).

The use of ICP in the context of CAR-T cells is addressed in a later paragraph.

CD70 blockers and antibody–drug conjugates (ADC) were also evaluated in NHL and MCL, such as SGN-CD70A, an ADC directed against the integral plasma membrane protein CD70. It has been evaluated in patients with DLBCL and MCL in a phase I trial that enrolled 20 patients. The ORR was 20%, including one complete response (CR), and the majority of responses were in patients with DLBCL. However, for now, the applicability was limited by the frequency and severity of thrombocytopenia (in 75% of the patients), despite the few long-term responses with limited drug exposure [91]. Moreover, SOX11 inhibitors are in preclinical development. Jatiani and colleagues reported that SOX11 inhibitors blocked the BCR signaling in SOX11-expressing MCL cells. Furthermore, these inhibitors display cytotoxic synergy with ibrutinib and induce cytotoxicity in SOX11-expressing ibrutinib-resistant MCL samples. These results provided a foundation for targeting SOX11 in MCL [92].

### 3.4. CAR-T Cells

#### 3.4.1. Initial Clinical Trials and Real-Life Results in MCL Third-Line Setting

CD19-directed CAR-T cells are now part of the therapeutic options for patients with R/R NHL including MCL, offering hope for curative responses thanks to a reprogramed T lymphocyte specificity and function able to target CD19 [93,94] (Table 3).

Brexucabtagene autoleucel (KTE-X19) has been evaluated in the ZUMA-2 phase II trial leading to FDA and EMA approval in 2020 for patients relapsing after two lines of treatments including prior chemotherapy with an anti-CD20 monoclonal antibody and a BTK inhibitor. Patients could receive bridging treatment to assure disease stability during the manufacturing process. In the intent-to-treat population (74 patients), the ORR was 85%, including 59% of CR [19,95]. At a median follow-up of 17.5 months, the 15-month PFS and OS rates were 59.2% and 76%, respectively [96].

In the real-life setting, data reported in two different large cohorts (one from the French LYSA group and one from the US CAR-T consortium) corroborated these results. Indeed, Herbaux and colleagues presented the results of KTE-X19 from patients included in the Descar-T French registry (LYSA Group). A total of 47 patients were infused with KTE-X19, of whom 42 patients had at least one efficacy evaluation. The ORR was 88%, including 61.9% of CR. CRS was noted in 78.7% of patients while neurotoxicity was observed in 48.9%. One patient died from a grade 5 CRS [97]. In the US real life experience from the lymphoma CAR-T consortium, 107 patients underwent leukapheresis, among whom 87% completed CAR-T cell infusion. The ORR was 86%, including 64% of CR. Interestingly, the ORR for a more aggressive form of MCL, i.e., blastoid or pleomorphic variants, was 94% with 70% of CR. The 3-month PFS was 80.6% and the 6-month OS rate was 82.1%. The CRS rate was 88%, and neurotoxicity was observed in 58% of patients. Twenty-four patients (26%) required ICU admission [98]. Lisocabtagene maraleucel (JCAR017) was also studied in patients with R/R MCL in the TRANSCEND NHL 001 phase II trial. The ORR was 84% (27/32 patients), including 59% of CR. Among the 12 patients with blastoid morphology, 7 patients (58%) achieved CR. Fifty percent of patients presented CRS (one grade 4). Neurologic events were present in nine patients (28%), including three patients (9%) with grade 3 [99].

**Table 3:** Results of CAR-T cells in MCL, in third line or more.

	Clinical Trials		Real Life Experience	
	ZUMA-2 [19,96]	TRANSCEND-001 [99]	Descar-T [97]	US Lymphoma CAR-T Consortium [98]
**CAR-T product**	brexucabtagene autoleucel (KTE-X19)	lisocabtagene maraleucel (JCAR017)	brexucabtagene autoleucel (KTE-X19)	brexucabtagene autoleucel (KTE-X19)
**N patients**	68	32	47	93
**N previous lines of TTT**	3	3	3	3
**Bridging, %**	37%	53%	87%	65%
**Blastoid or pleomorphic morphologic characteristics**	31%	37.5%	NR	45%
**Previous BTKi**	100%	87.5%	100%	82%
**ORR**	93%	84%	88%	86%
**CRR**	67%	59%	61.9%	64%
**ORR in blastoid**	93%	75%	NR	94% 70%
**PFS/DOR**	15-month PFS: 59.2% 15-month DOR: 58.6%	NR NR	Median PFS: 6.3 months	3-month PFS: 80.6%
**CRS**	Any grade: 91% Grade ≥ 3: 15%	Any grade: 50% Grade ≥ 3: 3%	Any grade: 78.7% Grade ≥ 3: 8.5%	Any grade: 88% Grade ≥ 3: 8%
**ICANS**	Any grade: 63% Grade ≥ 3: 31%	Any grade: 28% Grade ≥ 3: 9%	Any grade: 48.9% Grade ≥ 3: 8.5%	Any grade: 58% Grade ≥ 3: 33%
**Tocilizumab usage**	59%	31%	69.2%	76%

PFS: progression-free survival; ORR: objective response rate; CR: complete response; DOR: duration of response; CRS: cytokine release syndrome; ICANS: immune-effector-cell-associated neurotoxicity syndrome.

#### 3.4.2. Combination Strategies with or after CAR-T

Other strategies have been adopted to increase the efficacy of CAR-T cell therapy, especially combinations to foster the T cells and abrogate exhaustion with other agents (Table 4).

In the context of CAR-T cell failure in b-NHL, pembrolizumab has been evaluated with a disappointing ORR of 25% (3/12 patients, 1 with CR and 2 with PR) [100]. Nivolumab is currently under investigation in patients with hematologic malignancies who failed CAR-T cell therapy, including MCL (NCT04205409). In the combination setting, a phase II trial is evaluating the association of CD-19 CAR-T cells with acalabrutinib in patients with R/R MCL (NCT04484012). To deal with the failure related to target loss, autologous anti-CD20 CAR-T cell therapy (NCT03277729), but also bispecific anti-CD19/CD20 CAR-T cells, are under investigation in MCL (NCT04007029, NCT04186520). Autologous CAR-T (ATLCAR) cells targeted against the kappa light chain antibody are also being tested in a phase I trial of patients with kappa + NHL or CLL, including MCL (NCT04223765). As an example of many other strategies to improve efficiency, the TC-110 T cell, an autologous CAR with a single-domain antibody recognizing CD19 incorporated in the endogenous T-cell receptor (TCR) complex, is currently at the phase I/II trial stage of development (NCT04323657). Finally, allogenic CAR-T cells such as LUCAR-20S are also under investigation in a phase I trial in patients with R/R MCL (NCT04176913).

### 3.5. T-Cell-Engaging Bispecific Antibody

Bispecific T-cell engagers are artificial antibodies containing (at least) two different antigen-binding sites within one molecule, and simultaneously bind to an antigen on tumor cells and a molecule on the surface of T cells to induce tumor lysis [101]. Ongoing studies are highlighted in Table 5.

Blinatumomab is the first-in-class bispecific T-cell engager (BiTE) antibody targeting CD3/CD19 and is the most developed and the only one approved in B-cell malignancies. It has been approved by the FDA and EMA for the treatment of B-cell acute lymphoblastic leukemia (ALL). In a phase I trial of R/R B-cell NHL, Goebeler et al. reported an ORR of 69%, including 37% of CR. Twenty-four patients had R/R MCL [102]. On long-term follow-up, blinatumomab was associated with durable responses [103]. Interestingly, at target dose for efficacy, patients with R/R MCL had higher ORR than those with R/R DLBCL (71% vs. 55%, including 43% vs. 36% of CR/Cru, respectively). Neurological events were dose limiting, and the most significant toxicities were with 13 patients who discontinued treatment due to grade ≥ 3 adverse events [102]. These events as well as the continuous administration over 4 or 8 weeks hampered the development of the drug in NHL.

Mosunetuzumab is a humanized bispecific antibody targeting CD20 and CD3 and is evaluated in a first-in-human phase I/II trial as monotherapy or in combination with atezolizumab (PD-L1 inhibitor) in patients with R/R B-cell NHL and CLL (NCT02500407). The results of the phase I escalation study of single agent mosunetuzumab were recently reported. Two hundred thirty patients were enrolled. The best ORR was 34.9% in patients with aggressive B-cell NHL and 66.2% in those with indolent disease. Among patients who achieved CR, the median duration of response was 22.8 months in aggressive NHL and 20.4 months in indolent lymphomas. Mosunetuzumab was associated with a manageable safety profile. In the efficacy population, 13 patients had MCL with an ORR of 30.8% (4/13), including 23% of CR (3/13). Two patients presented stable disease and six patients experienced progressive disease as best response to treatment [104].

Glofitamab is a bivalent CD20-targeting T-cell-engaging bispecific antibody. The NP30179 phase I/II trial is currently studying glofitamab as monotherapy or in combination with obinutuzumab (single pretreatment dose) (NCT03075696). A pretreatment dose of obinutuzumab was administered in order to deplete peripheral and tissue-based B cells and mitigate serious CRS and was preferred over rituximab because of its deeper clearance of B cells [105,106]. In addition, the combination of T-cell therapy and IgG antibodies is an attractive approach due to the synergy of T-cell-mediated cytotoxicity and ADCC and phagocytosis induced by CD20-targeting antibodies [107,108]. Phillips and colleagues reported at the 2021 American Society of Hematology annual meeting the preliminary data from the NP30179 trial in patients with R/R MCL who received 1000 mg or 2000 mg of obinutuzumab pretreatment prior to glofitamab monotherapy. Twenty-nine patients were included in the analysis, with a median number of prior lines of three and of whom 69% had received prior BTKi therapy. The ORR was 81% (17/21 patients), including 66.7% of CR (14/21 patients). Similar response rates were reported in patients previously treated with BTK inhibitors or not. The most frequent adverse events were CRS (58.6%) followed by infusion-related reactions (24.1%). Neurologic adverse events occurred in 20.7% of patients [109]. Glofitamab is currently under evaluation in a phase II trial in patients with R/R B-cell NHL after progression on CAR-T cell therapy (NCT04703686).

Epcoritamab, is a CD3/CD20 BsAb with a subcutaneous administration. The results of a dose escalation cohort in a phase I/II trial of patients with R/R NHL (DLBCL and FL) was recently published. Epcoritamab was associated with an ORR of 68% in patients with R/R DLBCL (n = 46 patients) with 45% presenting a CR at full doses of 12–60 mg. Furthermore, the ORR was 90% in patients with R/R FL (n = 12 patients). The cohort included four patients with R/R MCL, with responses observed in two patients (50%), including one CR. Fever was the most common adverse event (69%) followed by cytokine release syndrome (59%), all grade 1 to 2, and injection site reactions (47%) [110]. The phase I/II trial is still ongoing (NCT03625037).

NVG-111 is a novel ROR1-CD3 bispecific antibody, targeting an oncofetal protein expressed in B-cell malignancies (mainly MCL and CLL, ALL) and acting as a receptor for the tumor growth factor Wnt5a, currently under evaluation in a first-in-human phase I trial in patients with ROR1-positive R/R MCL or CLL (NCT04763083).

## 4. Conclusions

In the past 10 years, impressive progress was made on the bed side for patients with MCL, leading to prolonged PFS and OS, but at the expense of toxicity with high-dose chemotherapy regimens and/or prolonged maintenance [111,112]. Tremendous progress on the bench side showed that TME has a key and central role in MCL tumor cell proliferation, but also that the B/TME interaction is fundamental in drug resistance. These discoveries led to the development of chemo-free treatments and combination strategies targeting both the tumor B cells and the TME. The field is currently moving and now focusing on novel unmet needs, as post CAR-T cell failure targeting the TME and both stromal and immune cells is doubtless crucial.

## Figures and Tables

**Table 2:** Immune checkpoint in MCL.

Trial	Population	Agents	Phase	N	Primary Endpoint
**NCT03153202**	R/R MCL or CLL	Pembrolizumab (anti-PD-1) + ibrutinib	I/II	40	DLT
**NCT03015896**	R/R B-cell NHL including MCL	Nivolumab + lenalidomide	I/II	102	Adverse events MTD
**NCT04599634**	R/R B-cell malignancies including MCL	Obinutuzumab with venetoclax and magrolimab (VENOM)	I	76	Safety and tolerability
**NCT02733042**	RR NHL and CLL. FUSION NHL 001	Durvalumab and Ibrutinib/Durva Benda	I/II	106	DLT

R/R: relapsed and/or refractory; MCL: mantle cell lymphoma; CLL: chronic lymphocytic leukemia; PD-1: programmed death-1; DLT: dose-limiting toxicities; NHL: non-Hodgkin lymphoma; MTD: maximal tolerated dose.

**Table 4:** Ongoing trials with CAR-T.

Trial	Population	Agents	Phase	N	Primary Outcome
**ZUMA-2 cohort 3 (NCT04880434)**	R/R MCL not receiving BTKi	brexucabtagene autoleucel (KTE-X19)	II	90	ORR
**TARMAC (NCT04234061)**	R/R MCL	tisagenlecleucel + ibrutinib	II	20	CR rate at month 4
**NCT04484012**	R/R MCL	CD19-targeting autologous CAR-T cell + acalabrutinib	II	36	CR rate DLT
**NCT03277729**	R/R B-cell NHL including MCL	CD20-targeting autologous CAR-T cell	I/II	35	DLT
**NCT04007029**	R/R B-cell NHL including MCL	bispecific Anti-CD19/CD20 autologous CAR-T Cells	I	24	Safety MTD
**NCT04186520**	R/R B-cell NHL and MCL	bispecific Anti-CD19/CD20 autologous CAR-T Cells	I/II	32	Number of adverse events
**NCT04176913**	R/R DLBCL, FL, MCL, and SLL	LUCAR-20S (CD20-targeting allogenic CAR-T cell)	I	34	DLT Adverse events Pharmacokinetics in blood and bone marrow
**NCT04223765**	kappa+ NHL or CLL including MCL	Autologous T Lymphocyte CAR cells targeting kappa light chain	I	20	Safety and tolerability
**NCT04323657**	R/R B-cell NHL including MCL and ALL	TC-110 (CD19 targeting TCR complex)	I/II	120	(RP2D) Efficacy in NHL and ALL

R/R: relapsed and/or refractory; MCL: mantle cell lymphoma; BTKi: Bruton’s tyrosine kinase inhibitor; NHL: non-Hodgkin lymphoma; DLBCL: diffuse large B-cell lymphoma; FL: follicular lymphoma; SLL: small lymphocytic lymphoma; CLL: chronic lymphocytic leukemia; ALL: acute lymphoblastic leukemia; TCR: T-cell receptor; CAR-T cells: chimeric antigen receptor T cells; DLT: dose-limiting toxicities; CR: complete response; ORR: objective response rate; RP2D: recommended phase 2 dose; N: number of patients; MTD: maximum tolerated dose.

**Table 5:** Ongoing studies with bispecific antibodies.

Study	Agent	Target	Phase	Nb of Patients	Population
**NCT04703686**	**Glofitamab (RO7082859) after single injection of obinutuzumab**	CD20x2/CD3	II	78	R/R lymphomas after failure of CAR-T cell
**NCT03467373**	**Glofitamab + R or O + CHOP or glofitamab + P + R + CHP**	CD20x2/CD3	Ib	172	R/R NHL or untreated DLBCL
**NCT03075696**	**Glofitamab as single agent or + O**	CD20x2/CD3	I/II	860	R/R B-cell NHL
**NCT03533283**	**Glofitamab + Atezolizumab or Polatuzumab Vedotin**	CD20x2/CD3	Ib/II	140	R/R B-cell NHL
**NCT05219513**	**Glofitamab + RO7443904**	CD20/CD3	I	200	R/R B-cell NHL
**NCT04082936**	**IGM-2323 (BsAb)**	CD20/CD3	I	160	R/R B-cell NHL (FL, DLBCL, MCL, MZL) after failure of at least 2 prior treatments
**NCT03625037**	**Epcoritamab (GEN3013)**	CD20/CD3	I/II	486	R/R B-cell lymphoma (DLBCL, PMBCL, FL, MCL, SLL, MZL)
**NCT04358458**	**Epcoritamab + GEN3009**	CD20/CD3 + CD37x2	I/II	182	R/R B-cell NHL
**NCT02924402**	**Plamotamab (XMAB13676)**	CD20/CD3	I	160	R/R non CLL B-cell malignancies
**NCT04763083**	**NVG-111**	ROR1/CD3	I/II	90	RR MCL or CLL, SLL
**NCT03888105 (ELM-2 trial)**	**Odronextamab (REGN1979)**	CD20/CD3	II	512 (78 MCL after BTKi failure)	R/R B-cell NHL including FL, DLBCL, MCL, MZL
**NCT02500407**	**Mosunetuzumab**	CD20/CD3	I/II	836	R/R B-cell NHL and CLL
**NCT03671018**	**Mosunetuzumab (BTCT4465A) + Polatuzumab Vedotin**	CD20/CD3	I/II	262	R/R FL, DLBCL, MCL

OS: overall survival; R/R: relapsed and/or refractory; CAR-T: chimeric antigen receptor T cells; NHL: non-Hodgkin lymphoma; FL: follicular lymphoma; DLBCL: diffuse large B-cell lymphoma; MCL: mantle cell lymphoma; MZL: marginal zone lymphoma; AEs: adverse events; PMBL: primary mediastinal B-cell lymphoma; SLL: small lymphocytic lymphoma; ORR: objective response rate; RP2D: recommended phase 2 dose; R: rituximab; O: obinutuzumuab; P: polatuzumab.
